# R_2_^2^(8) motifs in Aminopyrimidine sulfonate/carboxylate interactions: Crystal structures of pyrimethaminium benzenesulfonate monohydrate (2:2:1) and 2-amino-4,6-dimethylpyrimidinium sulfosalicylate dihydrate (4:2:2)

**DOI:** 10.1186/1752-153X-1-28

**Published:** 2007-11-13

**Authors:** Kasthuri Balasubramani, Packianathan Thomas Muthiah, Daniel E Lynch

**Affiliations:** 1 School of Chemistry, Bharathidasan University, Tiruchirappalli -620 024, Tamil Nadu, India; 2 Faculty of Health and Life Sciences, Coventry University, Coventry CV1 5FB, UK

## Abstract

**Background:**

Pyrimethamine [2,4-diamino-5-(*p*-chlorophenyl)-6-ethylpyrimidine] is an antifolate drug used in anti-malarial chemotherapy. Pyrimidine and aminopyrimidine derivatives are biologically important compounds owing to their natural occurrence as components of nucleic acids.

**Results:**

In the crystal structures of two organic salts, namely pyrimethaminium benzenesulfonate monohydrate **1 **and 2-amino-4, 6-dimethylpyrimidinium 3-carboxy-4-hydroxy benzenesulfonate dihydrate **2**, pyrimethamine (PMN) and 2-amino-4,6-dimethylpyrimidine (AMPY) are protonated at one of the nitrogens in the pyrimidine rings. In both the PMN and AMPY sulfonate complexes, the protonated pyrimidine rings are hydrogen bonded to the sulfonate groups, forming a hydrogen-bonded bimolecular ring motif with graph-set notation R_2_^2^(8). The sulfonate group mimics the carboxylate anion's mode of association, which is more commonly seen when binding with 2-aminopyrimidines. In compound **1**, the PMN moieties are centrosymmetrically paired through a complementary DADA array of hydrogen bonds. In compound **2**, two types of bimolecular cyclic hydrogen bonded R_2_^2^(8) motifs (one involving the carboxylate group and the other involving sulfonate group) coexist. Furthermore, this compound is stabilized by intra and intermolecular O-H...O hydrogen bonds.

**Conclusion:**

The crystal structures of pyrimethaminium benzenesulfonate monohydrate and 2-amino-4,6-dimethylpyrimidinium sulfosalicylate dihydrate have been investigated in detail. In compound 1, the R_2_^2^(8) motif involving the sulfonate group is present. The role the sulfonic acid group plays in mimicking the carboxylate anions is thus evident. In compound 2, two types of bimolecular cyclic hydrogen bonded R_2_^2^(8) motifs (one involving the carboxylate group and the other involving sulfonate group) coexist. In both the compounds base pairing also occurs. Thus homo and hetero synthons are present.

## Background

Intermolecular interactions and hydrogen bond motifs that occur repeatedly in crystal structures are called supramolecular synthons. Synthons are the recognition motifs between building blocks that can be used to propagate networks or supramolecular assemblies [1]. Hydrogen bonding patterns involving sulfonate groups in biological systems and metal complexes are of current interest [2-6]. Such interactions can be used for designing supramolecular architectures. Numerous hydrogen-bonding patterns of aminopyrimidine-carboxylate interactions [7,8] have been reported in the literature. Recently, different types of hydrogen-bonding motifs in sulfonate salts have been examined using the Cambridge Structural Database (CSD) [9].

The 2,4-diaminopyrimidine antifolates have been reviewed in the literature [10]. The different types of hydrogen-bonding patterns present in diaminopyrimidine-carboxylates [11] and aminopyrimidine complexes, such as pyrimethamine (PMN) hydrogen maleate, PMN hydrogen succinate, PMN hydrogen phthalate [12], PMN formate [13], PMN sulfosalicylate monohydrate [14], PMN *o*-nitrobenzoate, PMN *m*-nitrobenzoate, PMN *p*-nitrobenzoate [15], trimethoprim (TMP) benzenesulfonate monohydrate, TMP sulfanilate monohydrate, TMP *p*-toluene sulfonate and TMP sulfosalicylate dehydrate [16] 2-amino-4,6-dimethylpyrimidine (AMPY) hydrogen sulfate [17], 2-amino-4,6-dimethylpyrimidine-cinnamic acid [18], and 2-amino-4,6-dimethylpyrimidine-4-hydroxy benzoic acid [19] co-crystal structures, have been reported from our laboratory.

**Scheme 1:**
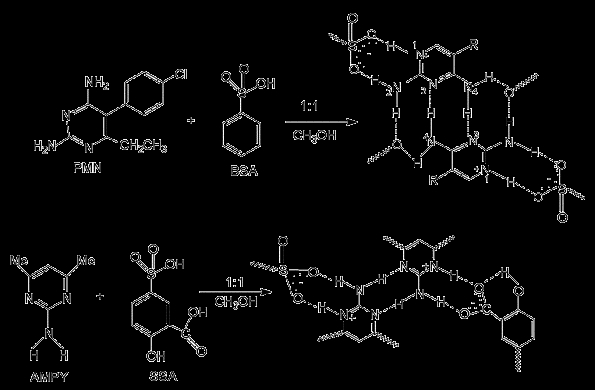
Hydrogen-bonded arrays and the supramolecular synthons present in the aminopyrimidine-sulfonate salts

Pyrimethamine [2,4-diamino-5-(*p*-chlorophenyl)-6-ethylpyrimidine] is an antifolate drug [20] used in anti-malarial chemotherapy. The drug binds with great affinity to the bacterial enzyme dihydrofolate reductase (DHFR) [21]. PMN is also used along with other drugs for the treatment of opportunistic infections in patients with AIDS [22]. 2-aminopyrimidine and its derivatives are of particular interest as adduct formers because of their ability to form stable hydrogen-bonded chains via their stereochemically associated amine group and the ring N atoms [23]. Hydrogen bonding plays a key role in molecular recognition [24] and crystal engineering [25]. The present study deals with hydrogen bonding, the nature of hydrogen-bonded arrays and the supramolecular synthons present in the aminopyrimidine-sulfonate salts (Scheme 1).

## Results and discussion

**Figure 1:**
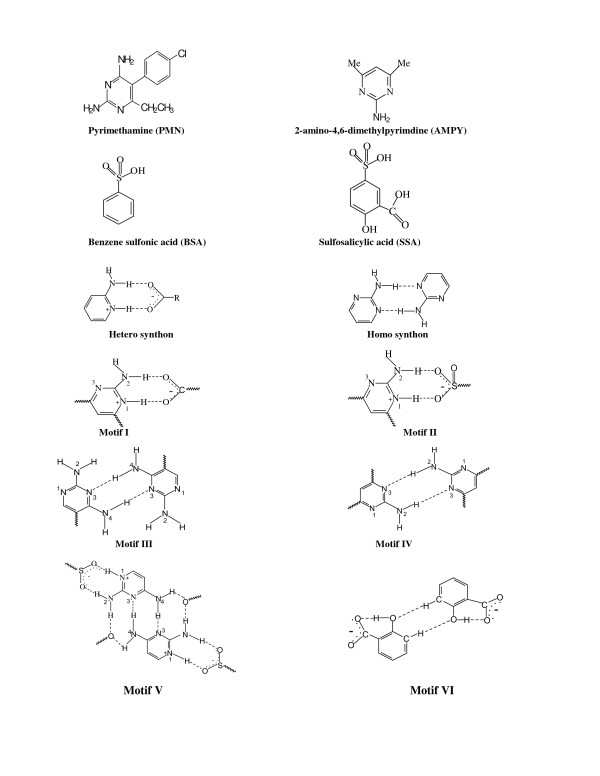
The schematic diagram for the various hydrogen-bonded motifs observed in compounds 1 and 2.

**Figure 2:**
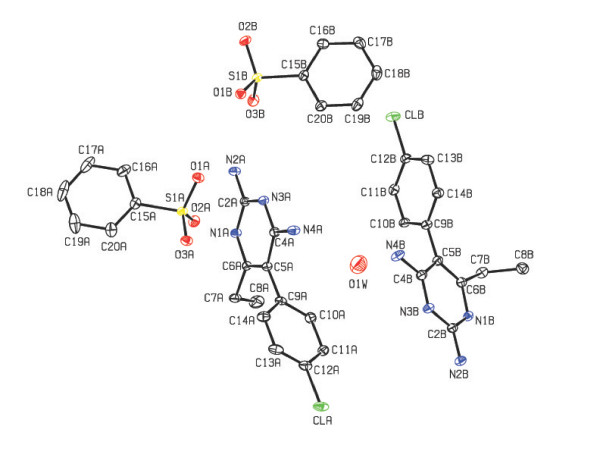
The ORTEP view of the asymmetric unit of the compound 1 (Hydrogen atoms are omitted for clarity).

**Figure 3:**
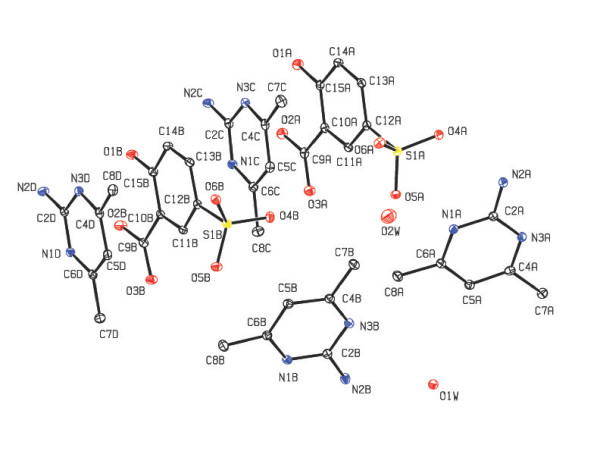
The ORTEP view of the asymmetric unit of the compound 2 (Hydrogen atoms are omitted for clarity).

**Figure 4:**
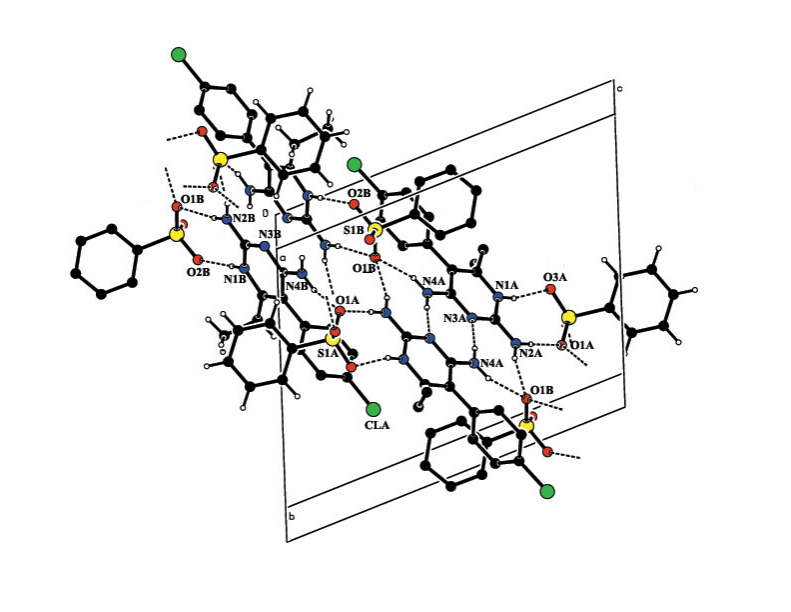
The hydrogen-bonded DADA array in the compound 1.

The three dimensional supramolecular architectures in such compounds can be analyzed in terms of various components such as motifs, chains, stacking interactions *etc*. The schematic representation of the hydrogen-bonded motifs observed in this study is shown in Figure 1. In the crystal structure of compound **1**, the asymmetric unit contains a pair of pyrimethaminium (PMN) cations (A and B), benzenesulfonate anions (A and B) and a water molecule as shown in Figure 2. In compound **2**, four molecules of 2-amino-4,6-dimethylpyrimidinium (AMPY) cations (A, B, C and D), two molecules of 3-carboxy-4-hydroxy benzenesulfonate (sulfosalicylate) anions (A and B), and two water molecules constitute the asymmetric unit (Figure 3). The PMN moieties are protonated at N1, leading to an enhancement of the internal bond angles at N1 [C2A-N1A-C6A 121.6(2)° and C2B-N1B-C6B 121.5(2)° in compound **1**]. The angles are larger than the values observed in neutral pyrimethamine [116.25(18)° (molecule A) and 116.09(18)° (molecule B)] [26]. In compound **2**, protonation of the pyrimidine base on the N1 site is reflected in the larger bond angle, as compared with the unprotonated site. The angles at the protonated N1 atom are C2A-N1A-C6A 121.85(17)°; C2B-N1B-C6B 121.93(17)°; C2C-N1C-C6C 121.64(18)° and C2D-N1D-C6D 121.73(18)°. The similar angles at the unprotonated N3 nitrogen are 117.20(17)° (molecule A), 117.52(17)° (molecule B), 117.35(18)° (molecule C) and 117.68(18)° (molecule D). The geometry of the pyrimidine cation agrees with that of other pyrimidine cations reported in the literature [27]. In compound **1**, the dihedral angles between the 2,4-diaminopyrimidine and the *p*-chlorophenyl are 78.50(12)° (molecule A) and 73.20(12)° (molecule B). These values are close to the values observed in the modelling studies carried out on the dihydrofolate reductase-pyrimethamine (DHFR-PMN) complexes [28]. The important torsion angles governing the orientation of the 6-ethyl group are C5A-C6A-C7A-C8A (-105.3(3)° in molecule A) and C5B-C6B-C7B-C8B (110.4 (3)° in molecule B). The lengths of the bonds connecting the pyrimidine and phenyl rings are 1.493(3)Å (molecule A) and 1.496(3)Å (molecule B). These values are in close agreement with those observed in the crystal structure of metoprine (1.495Å in molecule A and 1.478Å in molecule B) [29]. In compounds **1 **and **2**, the sulfonate group mimics the association of the carboxylate moiety and makes a hydrogen-bonded ring of graph-set notation R_2_^2^(8) with the PMN and AMPY cations. The hydrogen-bonding geometry of the N-donors to the sulfonate group gave a mean value for the N-H...O hydrogen bond distances involving sulfonates that was slightly longer in range when compared with the carboxylate O atoms indicating that sulfonates form longer and weaker hydrogen bonds with N-donors than carboxylates. These trends are also observed in the present investigation, as indicated in Table 1. The hydrogen bonds formed between the sulfonates and the N-donors were generally linear [30]. In compound **1**, the protonated pyrimethaminium (N1A and N1B) cations interact with the (O3A and O2B) oxygen atoms of the sulfonate anions through N-H...O hydrogen bonds (Heterosynthon) forming an eight membered ring motif R_2_^2^(8) [31-33] (motif II). The pyrimethaminium cations are centrosymmetrically paired through N4-H...N3 hydrogen bonds (Homosynthon) involving the 4-amino group and the N3 atom of the unprotonated pyrimidine to form the ring motif R_2_^2^(8) (motif III) (Table 1). In addition to the base pairing, one of the sulfonate oxygen atoms (O1B and O1A) (a hydrogen bond acceptor) bridges the 4-amino and the 2-amino groups on both sides of the pairing. The combination of such base-pairing patterns and the further bridging of the bases involved in the pairing by hydrogen bonds, leads to the formation of a linear array of four hydrogen bonds. This is called a complementary DADA array (motif V) of quadruple hydrogen-bonding patterns (D stands for hydrogen-bond donor, and A stands for hydrogen-bond acceptor). The corresponding graph-set notations are R_3_^2^(8), R_2_^2^(8) and R_3_^2^(8) (Figure 4). This type of DADA array of quadruple hydrogen bonds has been observed in some previously reported crystal structures [12].

**Table 1:** Hydrogen bonding geometry for the compounds 1 and 2

Compound	D-H...A	H...A	D...A	D-H...A
**1**.	N1A – H1A .. O3A	1.90	2.733(3)	164
	N1B – H1B .. O2B^a^	1.89	2.742(3)	169
	N2A – H3A .. O1B	2.15	2.980(3)	161
	N2B – H3B .. O2A^b^	2.00	2.843(3)	167
	N2A – H4A .. O1A	1.99	2.847(3)	175
	N2B – H4B .. O1B^a^	2.23	3.076(3)	168
	N4A – H5A .. N3A^c^	2.16	3.013(3)	170
	N4A – H6A .. O1B^c^	2.33	3.015(3)	137
	N4B – H6B .. O1A^c^	2.43	3.079(3)	133
	C10B – H10B .. O2A^d^	2.54	3.364(4)	148
				
**2**.	N1B–H1B1 · · · O6B^e^	1.84	2.693 (2)	168
	O1A–H1A · · · O2A	1.81	2.539 (2)	147
	O1B–H1B · · · O2B	1.77	2.498 (2)	147
	N1C–H1C · · · O2B^f^	1.75	2.614 (2)	179
	N1D–H1D · · · O2A^f^	1.83	2.682 (2)	174
	O1W–H1W · · · O5B^g^	1.97	2.785 (2)	139
	N1A–H1A1 · · · O5A	1.87	2.722 (2)	168
	O1W–H2W · · · O6A^e^	2.08	2.775 (2)	127
	N2C–H2C1 · · · O3B^f^	2.05	2.908 (2)	173
	N2C–H2C2 · · · N3D^h^	2.16	3.010 (3)	169
	N2A–H2A1 · · · O1W^i^	2.10	2.927 (2)	162
	N2A–H2A2 · · · O4A	2.03	2.889 (2)	172
	N2D–H2D1 · · · N3C^j^	2.16	3.003 (3)	165
	N2D–H2D2 · · · O3A^f^	1.95	2.809 (2)	173
	N2B–H2B1 · · · O1W	2.10	2.933 (2)	164
	N2B–H2B2 · · · O4B^e^	2.01	2.871 (2)	177
	C5A–H5A · · · O4A^e^	2.51	3.382 (2)	156
	C5B–H5B · · · O4B	2.51	3.352 (2)	151
	C5B–H5B · · · O5B	2.58	3.428 (3)	152
	C11A–H11A · · · O5A	2.43	2.849 (2)	107
	C11B–H11B · · · O5B	2.60	2.946 (2)	103
	C8D–H8D2 · · · O6B	2.47	3.378 (3)	159
	C14B–H14B · · · O1B^h^	2.36	3.275 (3)	169
	C7B–H7B3 · · · O5A	2.52	3.421 (3)	156
	C7A–H7A1 · · · O6B^j^	2.50	3.458 (3)	174
	C7D–H7D3 · · · O3B	2.58	3.413 (3)	145

(a) x-1, y-1, z-1; (b) x, y-1, z-1; (c) 2 - x, 1 - y, 1 - z; (d) 1-x, 1-y, 1-z ; (e) x-1, y, z; f) 1-x, 1-y,-z; (g) 1-x, 1-y, 1-z; (h) 2-x, 1-y,-z; (i)1 - x,-y, 1 - z; (j) x - 1, y - 1, z.

**Figure 5:**
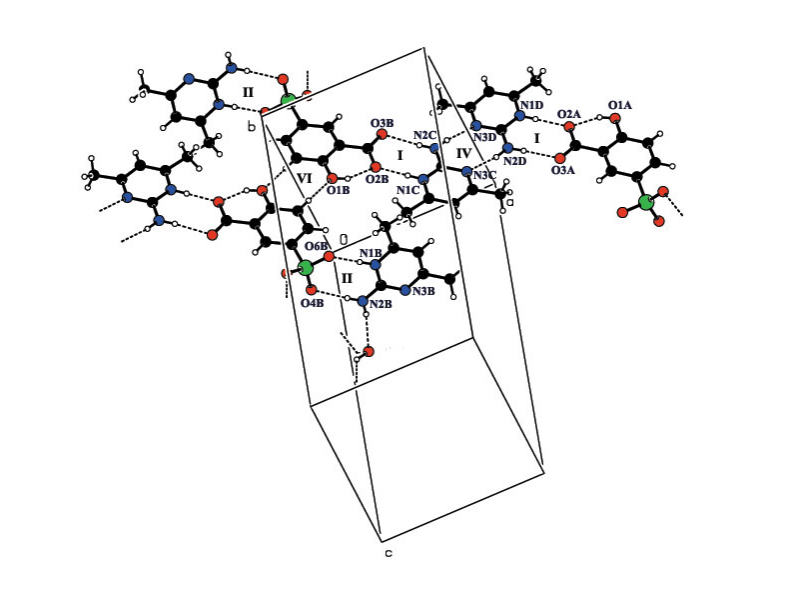
Hydrogen-bonding patterns involving carboxylate/sulfonate groups in compound 2 **(I, II, IV and VI indicates hydrogen bonded motifs)**.

**Figure 6:**
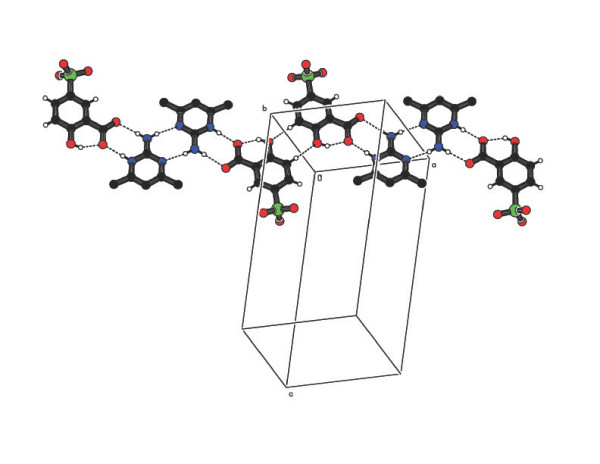
One dimensional chain made up of sulfosalicylate and AMPY molecule in compound 2.

**Figure 7:**
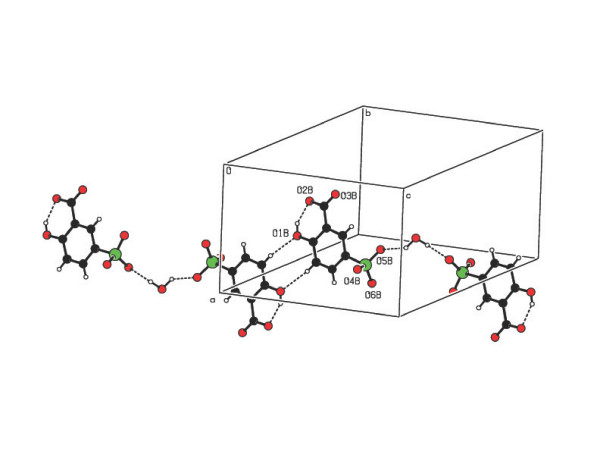
A view of the supramolecular chain made up of sulfosalicylate and water molecule in compound 2.

**Figure 8:**
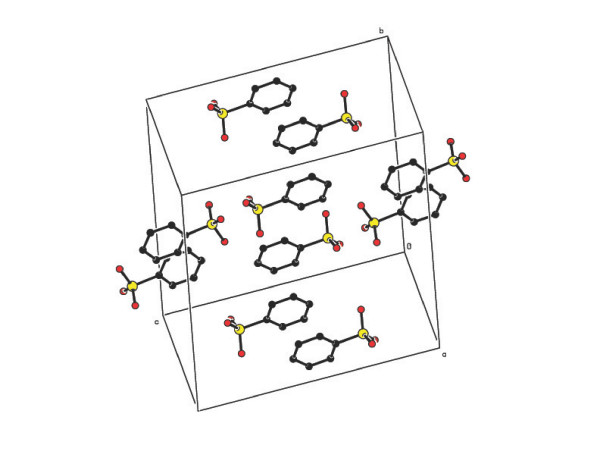
A view of π-π stacking interaction between benzenesulfonate anions in compound 1.

**Figure 9:**
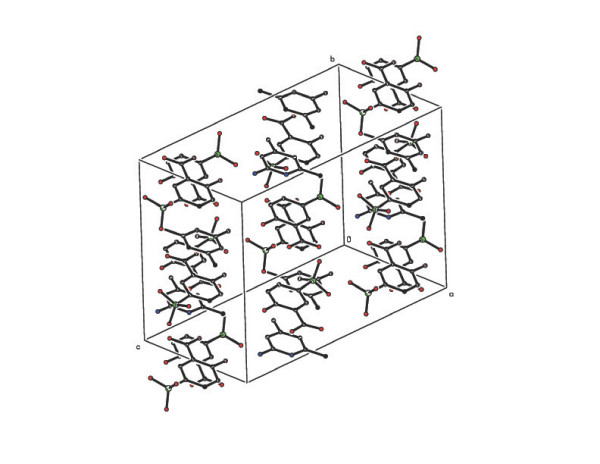
π-π stacking interactions in compound 2.

In compound **2**, two types of bimolecular cyclic hydrogen-bonded R_2_^2^(8) motifs (motif I and motif II) are formed. Motif I involves protonated aminopyrimidinium cations (N1A and N1B), and the 2-amino group and sulfosalicylate anions (carboxylate group) (O5A and O6B). Motif II is formed by protonated aminopyrimidinium cations (N1C and N1D), and the 2-amino group and sulfosalicylate anions (sulfonate group) (O2B and O2A). There is also base pairing via a pair of N-H...N hydrogen bonds (motif IV) involving two aminopyrimidinium molecules (cations C and D). These arrays are connected *via *a pair of C-H...O hydrogen bonds involving centrosymmetrically paired sulfosalicylates (B molecule) to form a supramolecular network (Figure 5). The commonly observed intramolecular hydrogen bond between the phenol -OH and carboxyl group in salicylic acid is also present in the sulfosalicylate anion (motif VI) [32]. The two sulfosalicylate (carboxylate group) oxygen atoms interact with 2-amino-4,6-dimethylpyrimidinium cations through C-H...O hydrogen bonds. The base pairing and C-H...O hydrogen bonds are arranged alternatively to form a chain (Figure 6). The two sulfosalicylate anions (O5B and O6A) are bridged by the water molecule (O1 W) via O-H...O hydrogen bonds (Figure 7). In compound **1**, PMNBSA, π-π stacking interactions between benzenesulfonate molecules are observed with a perpendicular separation of 3.356Å, a centroid-to-centroid distance of 3.608(2) Å and a slip angle of 21.54° (Figure 8). In compound **2**, AMPYSSA, the 2-amino-4,6-dimethylpyrimidinium cations (C), stack with sulfosalicylate anions A and B, with a perpendicular separation of 3.319Å and 3.359Å, a centroid-to-centroid distance of 3.529(11)Å and 3.554(11)Å and a slip angle of 17.49° and 19.30° respectively. A similar type of stacking is also observed between the 2-amino-4,6-dimethylpyrimidinium cation (D) and the sulfosalicylate anions (A and B), with a perpendicular separation of 3.238Å and 3.360Å, a centroid-to-centroid distance of 3.730(11)Å and 3.483(11)Å and a slip angle of 24.13° and 19.30° respectively (Figure 9). These are typical aromatic stacking values [34].

## Conclusion

The crystal structures of pyrimethamine benzenesulfonate monohydrate and 2-amino-4,6-dimethylpyrimidine sulfosalicylate dihydrate have been investigated in detail. In compound 1, the R_2_^2^(8) motif involving the sulfonate group is present. The role the sulfonic acid group plays in mimicking the carboxylate anions is thus evident. In compound 2, two types of bimolecular cyclic hydrogen bonded R_2_^2^(8) motifs (one involving the carboxylate group and the other involving sulfonate group) coexist. In both the compounds base pairing also occurs. Thus homo and hetero synthons are present. These synthons combine to form a supramolecular network. This observation is relevant for many supramolecular architectures and crystal engineering.

### Experimental

Compounds **1 **and **2 **were prepared by the mixing of hot methanolic solutions of PMN (62 mg, Shah Pharma-chem, India) or AMPY (Aldrich) and the corresponding acids -benzene sulfonic acid (40 mg, Merck) & 3-carboxy-4-hydroxy-benzene sulfonic acid (55 mg, Merck) in 1:1 molar ratio and warming over water bath for 20 min. After a few days blocks of colourless crystals of compound **1 **and **2 **were obtained.

### X-ray Crystallography

X-ray diffraction data were collected on a Bruker Nonius Kappa CCD area detector diffractometer by using MoKα (λ 0.71073 Å) for compounds **1 **and **2 **at 120(2)K. The structures were solved by direct methods and refined by full-matrix least squares (on F^2^) SHELXS97 and SHELXL97 [34], with the graphics produced using PLATON97 [35]. All the non-hydrogen atoms were located from a Fourier map and refined anisotropically. All the hydrogen atoms for compounds **1 **and **2 **were positioned geometrically and refined as riding. In compound **1**, the O1 W of water is disordered and the hydrogen atoms are not found. In compound **2**, the water (O1 W) hydrogen atoms are located from difference Fourier map. The O2 W of water is disordered, the hydrogen atoms are not found. The crystal data and details of structural determination for the compounds 1 and 2 are given in Table 2. CCDC reference numbers: 611485 and 611479. All CIF information can be found in Additional file 1.

**Table 2:** Crystallographic parameters for 1 and 2

Properties	1	2
Formula	2(C_12_H_14_Cl N_4_),	4(C_6_H_10_N_3_),
	2(C_6_H_5_O_3_S), O0.41	2(C_7_H_4_O_6_S), H_2_O, O
M.wt	820.34	963.04
Crystal System	Triclinic	Triclinic
Space group	P-1	P-1
a/A°	10.2783(3)	9.2466(2)
b/A°	13.6919(3)	14.0976(3)
c/A°	15.4164(4)	17.6365(4)
α/°	102.863(2)	94.0280(10)
β/°	102.187(2)	101.1930(10)
γ/°	108.805(2)	91.9680(10)
V/A°^3^	1905.83(10)	2247.00(9)
Z	2	2
Radiation λ/A°	0.71073	0.71073
Dc/g cm^-3^	1.429	1.423
T/K	293(2)	293(2)
μ/mm^-1^	0.338	0.198
F(000)	855	1012
Reflection collected	8768	10346
Observed data [I>2σ(I)]	5759	8165
Parameters refined	498	606
Final R_1 _on observed data	0.0509	0.0605
Final wR_2 _on observed data	0.1314	0.1555
Structure solution	SHELXS97 [36]	SHELXS97
Structure refinement	SHELXL97	SHELXL97
Graphics	PLATON97 [37]	PLATON97

## Authors' contributions

This work was prepared in the research group of PTM. He proposed the work and drafted the manuscript. KB participated in the design and presiding the experiments and drafted the manuscript. DEL collected the X-ray data and drafted the manuscript.

## Supplementary Material

Additional file 1 Crystallographic Information. Contains all relevant CIF information. Click here for file
